# Mitochondrial connection to Alzheimer’s disease and heart failure

**DOI:** 10.1016/j.cophys.2025.100830

**Published:** 2025-05-10

**Authors:** Anupriya Sinha, Natasha Jaiswal, Pooja Jadiya, Dhanendra Tomar

**Affiliations:** 1 Department of Cardiovascular Medicine, Wake Forest University School of Medicine, Winston-Salem, NC 27157, USA; 2 Department of Internal Medicine, Section of Gerontology and Geriatric Medicine, Wake Forest University School of Medicine, Winston-Salem, NC 27157, USA; 3 Sticht Center for Healthy Aging and Alzheimer’s Prevention, Wake Forest University School of Medicine, Winston-Salem, NC 27157, USA

## Abstract

The brain and heart are intricately linked, with dysfunction in one organ often affecting the other. Cardiovascular diseases (CVDs), particularly heart failure, impair cerebral blood flow, contributing to cognitive decline and increasing dementia risk. Conversely, Alzheimer’s disease (AD), marked by amyloid-beta plaques and tau tangles, impacts cardiac function. A shared mechanism between AD and CVDs is mitochondrial dysfunction, which disrupts energy production and oxidative balance, worsening both neurodegeneration and heart health. This interdependence underscores the potential for mitochondria-targeted therapies to address both conditions. With an aging population facing rising incidences of AD and CVDs, understanding these interconnected pathways and the central role of mitochondria could inform new therapeutic strategies and improve outcomes in both neurodegenerative and cardiovascular diseases.

## Introduction

The heart and brain are closely connected, with dysfunction in one often affecting the other. Cardiac dysfunction can lead to cognitive decline and neurodegeneration, while brain disorders like Alzheimer’s disease (AD) can negatively impact cardiovascular health. This bidirectional link highlights the strong relationship between cardiovascular diseases (CVDs) and neurodegenerative conditions [1]. Reduced heart function can lower cerebral blood flow, depriving the brain of oxygen and essential nutrients, which can trigger cognitive decline and elevate dementia risk. Conditions like atrial fibrillation (AF) and chronic heart failure are strongly linked to neurological deterioration. Additionally, mutations in presenilin genes (PSEN1, PSEN2), key in Alzheimer’s, are also associated with cardiac disorders such as dilated (DCM) and hypertrophic cardiomyopathies (HCM), revealing shared genetic and pathological pathways [2,3].

**Figure 1:**
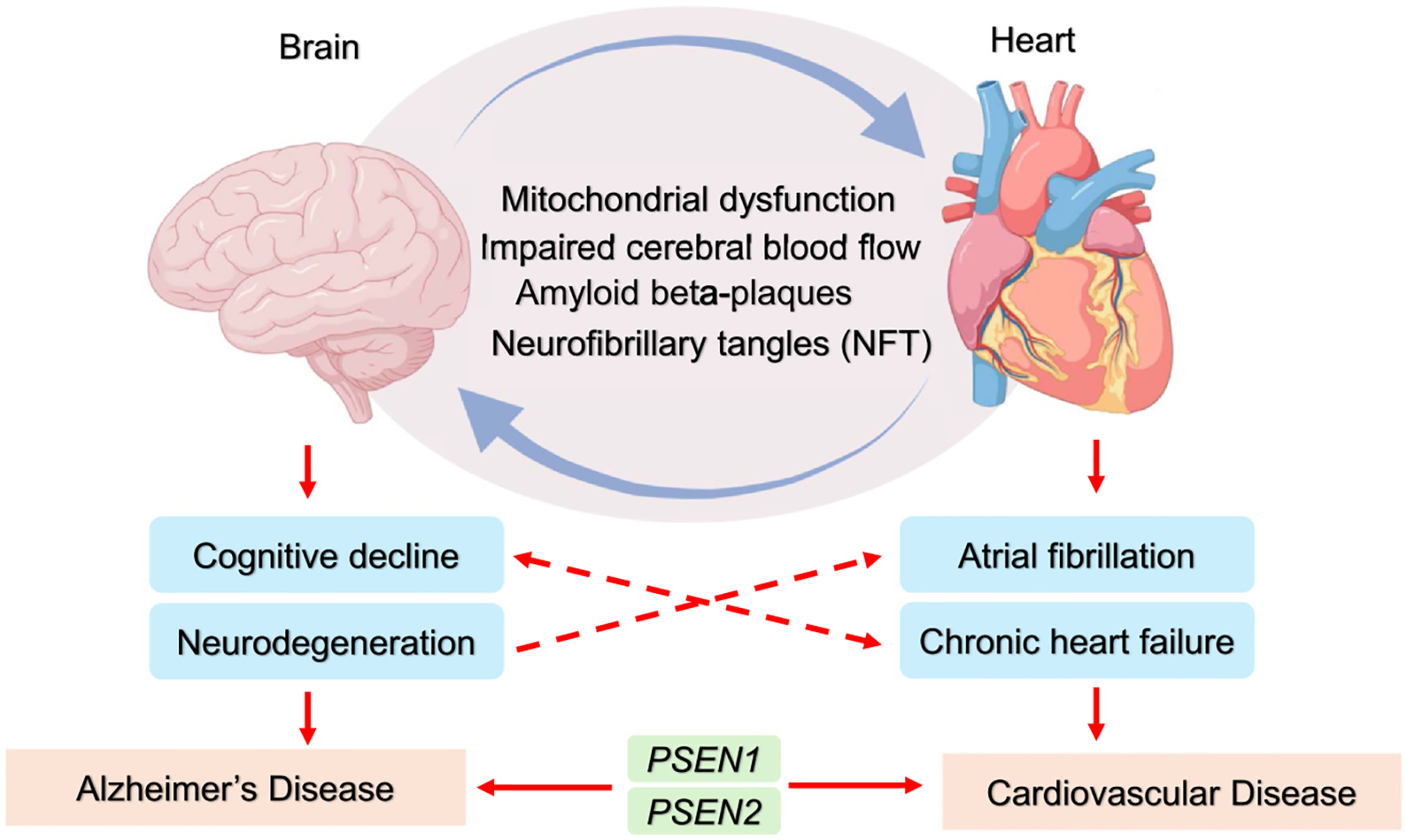
Bidirectional relationship between Alzheimer’s Disease (AD) and cardiovascular diseases (CVDs) through mitochondrial dysfunction. This figure illustrates the shared pathologies between AD and CVDs, highlighting mitochondrial dysfunction as a central contributor to both conditions. Hallmark features of AD, including Aβ plaque accumulation and neurofibrillary tangles, PSEN mutations, lead to neuronal damage and are found in cardiac dysfunctions. Cardiac dysfunction leads to cerebral hypoperfusion, depriving the brain of oxygen and nutrients, and accelerating cognitive decline.

AD is a neurodegenerative condition marked by dementia and cognitive decline. Its key features include amyloid-β (Aβ) plaque buildup and neurofibrillary tangles from hyperphosphorylated tau, leading to neuronal loss and brain atrophy [4]. In addition to these, mitochondrial dysfunction, vascular issues, oxidative stress, and neuroinflammation also drive disease progression [5,6]. The two-way link between AD and CVDs is underscored by shared proteotoxicity. Aβ and tau aggregates, which drive neuronal damage in AD, also impair heart muscle cells, causing issues like diastolic dysfunction. In turn, cardiac dysfunction worsens cognitive decline by limiting brain blood flow, forming a vicious cycle that accelerates AD progression (Figure 1).

Mitochondrial dysfunction is a key factor connecting AD and CVDs. As the cell’s energy centers, mitochondria are essential for ATP production, oxidative stress regulation, and apoptosis control. In AD, impaired mitochondria reduce energy supply and increase oxidative damage, driving neuronal loss [7–9]. Similarly, mitochondrial defects in heart cells contribute to reduced cardiac function and heart failure [10,11]. This review discusses the latest research on AD and CVDs, emphasizing how cardiac dysfunction and cognitive decline are linked to mitochondrial health.

## Crosstalk between cardiac and brain dysfunction in Alzheimer’s and cardiovascular diseases

Cognitive decline and the onset of dementia, particularly AD, have traditionally been linked to brain-specific pathologies. However, recent studies emphasize the role of systemic dysfunction, particularly cardiovascular health, in exacerbating these conditions. For instance, research has shown that AD patients often exhibit diastolic dysfunction [12], impairing the heart’s capacity to pump blood effectively during relaxation and contributing to cerebral hypoperfusion. This reduced blood flow can accelerate cognitive decline, creating a direct link between cardiac health and neurodegenerative processes [13]. Moreover, the severity of heart failure has been found to positively correlate with the degree of cognitive impairment, supporting the view that cardiac dysfunction significantly influences the progression of diseases like AD [13].

Compromised cerebral blood flow in CVD patients creates a cascade of metabolic disruptions, such as acidosis and oxidative stress, that lead to neuronal degradation [14]. Structural brain changes, such as atrophy and demyelination, have been noted in heart failure patients, suggesting that these cardiac abnormalities can directly impair the brain’s ability to maintain cognitive functions [15]. Notably, AF, a prevalent cardiac arrhythmia, has been shown to accelerate cognitive decline. The Intermountain Heart Collaborative Study revealed that AF significantly increases the risk of dementia, including AD, even in individuals younger than 70 years old [16]. AF’s irregular blood flow increases the likelihood of silent strokes and microinfarcts, compounding the risk of cognitive deficits. Furthermore, comorbid conditions such as hypertension, obesity, and diabetes amplify these effects, worsening brain vasculature and neuronal network integrity and elevating the risk of dementia [16–18]. Thus, cardiovascular dysfunction not only impacts the heart but also plays a significant role in accelerating AD progression by compromising cerebral metabolism and structural health.

Evidence shows that cardiac dysfunction contributes to cognitive decline and aggravates underlying AD pathology through shared molecular mechanisms. One such mechanism is protein aggregation, which is central to both cardiac and brain dysfunctions [18]. In AD, the accumulation of Aβ peptides and tau proteins leads to neuronal cell death, inflammation, and impaired calcium homeostasis. Similarly, protein misfolding and proteotoxicity in the heart can lead to conditions such as DCM, HCM, and heart failure. Intriguingly, Aβ deposits, long regarded as markers of AD in the brain, have also been found in the myocardium of patients with idiopathic DCM [19]. These cardiac Aβ aggregates disrupt calcium regulation and promote cell death, mirroring their harmful effects in the brain [12].

Genetic studies further highlight the link between heart and brain dysfunction. Mutations in the PSEN1 and PSEN2 genes, implicated in familial AD, have also been identified in patients with familial DCM and heart failure [3]. These mutations can lead to aberrant protein aggregation, and in some cases, both Aβ and amylin deposits have been found in the brains and hearts of AD patients, suggesting a systemic proteotoxic process that impacts multiple organs [20]. Additionally, Aβ deposition in cardiac tissue and amyloid buildup in blood vessels contribute to arterial stiffness and atherosclerosis, worsening cardiac function, and amplifying AD progression. Cardiac conditions like AF can exacerbate cerebral amyloid angiopathy by promoting Aβ deposition in cerebral vessels, further impairing cognitive function [20,21].

This compelling evidence of the heart–brain crosstalk in both cardiac dysfunction and AD pathology emphasizes the importance of cardiovascular health in the management and progression of neurodegenerative diseases. Understanding these interactions can guide more integrated therapeutic strategies aimed at mitigating the dual burden of CVDs and AD.

## Mitochondrial dysfunction: a common mechanism in heart and brain diseases

Mitochondrial dysfunction serves as a common pathological link between CVDs and AD through interconnected pathways involving oxidative stress, inflammation, metabolic dysregulation, and impaired mitochondrial quality control. Excessive reactive oxygen species (ROS) generation leads to lipid peroxidation, protein misfolding, and DNA damage, which contributes to synaptic loss in AD and endothelial dysfunction in CVDs [7,8,22]. Inflammatory signaling is another key contributor, with mitochondrial antiviral-signaling protein (MAVS) playing a role in both neuroinflammation and vascular inflammation, promoting chronic damage in both conditions [23]. Additionally, AMP-activated protein kinase (AMPK) dysfunction disrupts energy homeostasis, exacerbating neuronal loss in AD and endothelial stress in CVDs [24,25]. Moreover, mitochondrial DNA released into circulation acts as a damage-associated molecular pattern, activating immune responses that further amplify neurodegeneration and vascular disease progression [26,27].

Mitochondria constantly undergo fusion and fission to maintain their function and quality [28]. However, in both AD and CVDs, these processes become dysregulated, leading to excessive fission and mitochondrial fragmentation and impairing their function [28,29]. In desmin-related cardiomyopathy, this fragmentation reduces ATP production and increases the likelihood of cardiomyocyte death, contributing to heart failure [30]. Similarly, in AD, increased mitochondrial fission driven by the upregulation of proteins like dynamin-related protein 1 (Drp1) results in impaired energy production and increased oxidative stress, promoting neurodegeneration [31,32]. Mitochondrial respiratory chain defects, particularly in complex I and complex IV of the electron transport chain (ETC), are common in both diseases. These defects lead to reduced ATP production and increased production of ROS, which cause further cellular damage [31–33]. In AD, impaired mitochondrial respiration accelerates neuronal death and cognitive decline [34,35]. Likewise, in cardiomyopathies, mitochondrial dysfunction leads to reduced heart muscle contractility and contributes to the progression of heart failure [36]. The shared vulnerabilities of mitochondrial energy failure in both the heart and brain underscore their susceptibility to systemic metabolic disruptions.

Insights from models like the CryAB^R120G^ mouse, which carries a mutation in the α-B-crystallin gene linked to protein misfolding and mitochondrial dysfunction, reveal that impaired oxidative phosphorylation contributes to both cardiac and neurological deterioration [37]. These models show mitochondrial dysfunction in cardiac cells, leading to severe heart failure. Interestingly, similar defects are observed in the brain, where impaired oxidative phosphorylation is linked to Alzheimer’s-like symptoms, such as cognitive decline and Aβ accumulation [34,35]. This suggests a shared mitochondrial pathway underlying dysfunction in both the heart and brain.

A key driver of this shared dysfunction is oxidative stress. In AD, oxidative stress accelerates the formation of Aβ plaques and tau tangles, exacerbating neuronal damage. In cardiomyopathies, oxidative stress damages cardiomyocytes, contributing to heart failure. This shared oxidative stress between the heart and brain creates a vicious cycle that amplifies mitochondrial dysfunction and further deteriorates both organs [36]. Another critical factor is mitochondrial calcium dysregulation. Mitochondria absorb calcium to stimulate ATP production, but excessive calcium uptake can lead to mitochondrial overload, triggering apoptosis. We and others have reported that dysregulated mitochondrial calcium signaling is an early and significant contributor to AD initiation and progression [2,13,16,36] [7–9]. Similarly, in cardiomyopathies, calcium dysregulation leads to impaired heart muscle contraction and cell death, further worsening heart failure [38]. The opening of the mitochondrial permeability transition pore (mPTP) due to calcium overload accelerates cell death in both heart and brain, magnifying the cycle of oxidative stress, energy deficits, and disease progression [39,40].

Further compounding this dysfunction is the disruption of mitochondrial–nuclear crosstalk, which is essential for maintaining cellular homeostasis and varies across cell types and brain regions [41]. In AD, impaired mitonuclear signaling, especially in synaptic and lysosomal pathways, disrupts energy balance and undermines mitochondrial quality control. Mitochondrial stress activates retrograde signaling, including the mitochondrial unfolded protein response and the integrated stress response, which affect proteostasis and cell survival [42]. Similarly, in CVDs, defective mitonuclear communication contributes to metabolic imbalance, oxidative damage, and chronic inflammation, driving disease progression [43].

**Figure 2:**
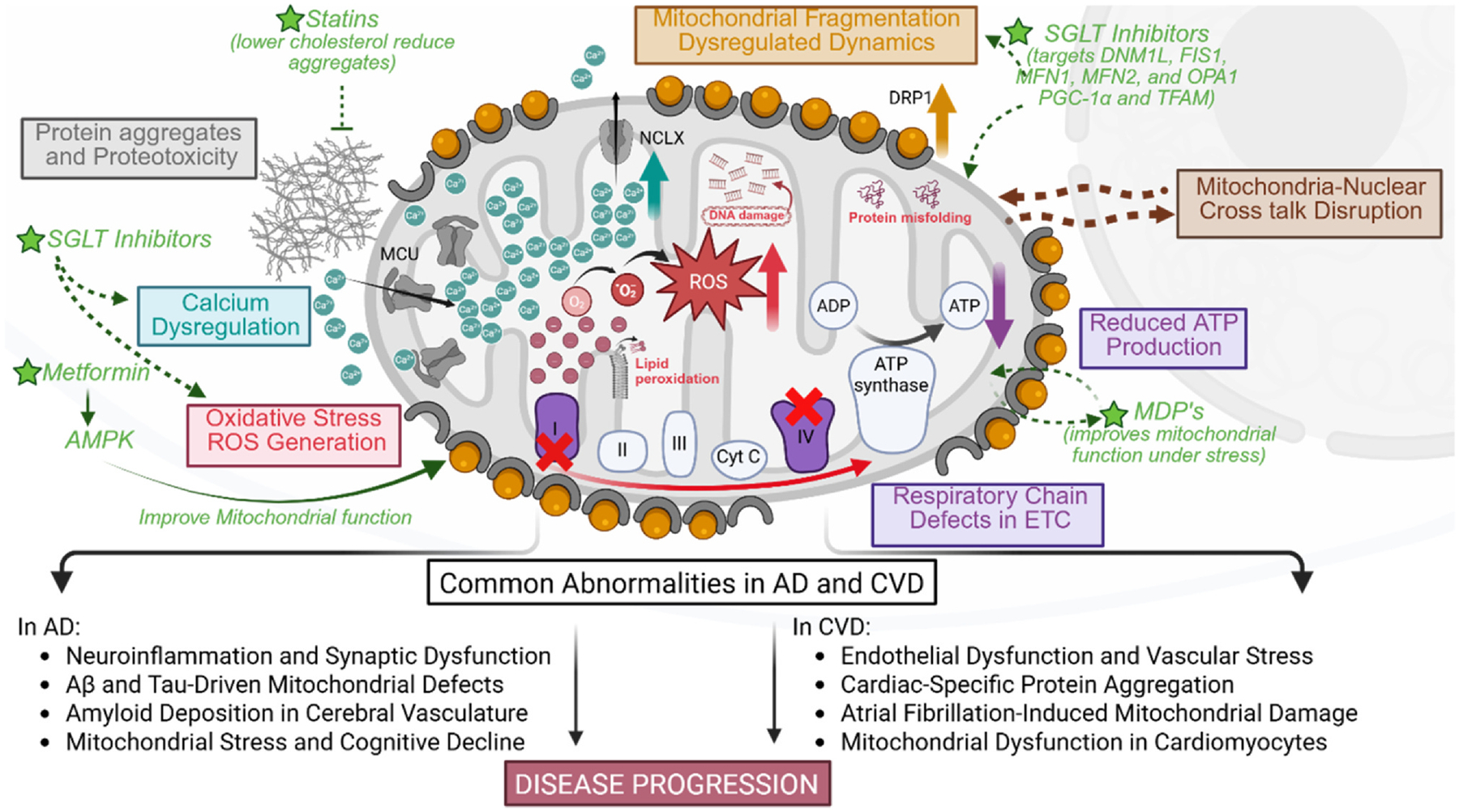
Mitochondrial dysfunction in Alzheimer’s disease (AD) and cardiovascular disease (CVD). This figure illustrates key mitochondrial abnormalities common to both AD and CVD such as; Oxidative stress and excessive reactive oxygen species (ROS) generation (in red), which contribute to mitochondrial damage, lipid peroxidation, and DNA mutations, exacerbating neurodegeneration and cardiovascular dysfunction. Respiratory chain defects in the electron transport chain (ETC), particularly in Complex I and IV, reduced ATP production (in purple) and increased oxidative stress, leading to metabolic insufficiency. Calcium dysregulation disrupts mitochondrial homeostasis, triggering calcium overload (in blue), mitochondrial permeability transition pore (mPTP) opening, apoptosis, and impaired cellular energy balance. Additionally, mitochondria–nuclear crosstalk disruption affects transcriptional responses, further impairing cellular function (in brown). Accumulation of protein aggregates, such as amyloid-β (Aβ) and tau, contributes to proteotoxicity and mitochondrial dysfunction (in gray). Furthermore, dysregulated mitochondrial dynamics, driven by increased Dynamin-related protein 1 (Drp1) activity (in orange), lead to excessive mitochondrial fragmentation, reducing mitochondrial connectivity and cellular function. These common abnormalities further lead to cell- and organ-specific aberrations driving disease progression. Potential therapeutics targeting mitochondrial dysfunction to improve AD and CVD include statins, Sodium-Glucose Cotransporter (SGLT) inhibitors, mitochondrial-derived peptides (MDPs), and Metformin, which by reducing oxidative stress and improving mitochondrial function through different molecular pathways highlighted in this figure possess neuroprotective and cardioprotective roles.

Taken together, mitochondrial dysfunction emerges as a unifying mechanism in heart–brain crosstalk, linking AD and CVDs through shared pathways of energy deficiency, oxidative stress, calcium imbalance, and impaired signaling. This connection underscores the importance of targeting mitochondrial health to break the feedback loop of mutual deterioration between heart and brain (Figure 2). The overlapping mechanisms of cardiac and neurodegenerative disorders, summarized in Table 1, include diastolic dysfunction, cerebral hypoperfusion, AF, protein aggregation, and mitochondrial impairment, pointing to promising shared therapeutic targets.

## Conclusion and future directions

Mitochondrial dysfunction clearly emerges as a central, shared mechanism linking cardiac disorders and neurodegenerative diseases such as AD and cardiomyopathies. This intersection is driven by overlapping pathological pathways, including impaired oxidative phosphorylation, disrupted mitochondrial dynamics, calcium imbalance, elevated oxidative stress, and defective mitochondrial–- nuclear communication. In addition, shared genetic mutations and systemic protein aggregation reinforce the deep biological connection between heart and brain pathologies. These findings collectively position mitochondria as a strategic therapeutic target for slowing or preventing the progression of both cardiovascular and neurodegenerative diseases. Several existing drugs already target pathways common to both AD and CVD.

Statins, for example, not only lower cholesterol but also reduce amyloid plaque formation and atherosclerosis risk [44,45]. Metformin enhances mitochondrial function and lowers insulin resistance, while sodium-glucose cotransporter 2 (SGLT2) inhibitors improve mitochondrial efficiency and reduce the risk of heart failure [46]. Cholinesterase inhibitors, commonly used in AD, enhance acetylcholine signaling, benefiting both cognitive function and vascular health. Similarly, angiotensin receptor blockers lower blood pressure and provide neuroprotective effects against stroke and neurodegeneration. Non-steroidal anti-inflammatory drugs help curb inflammation, slowing both amyloid accumulation and cardiovascular damage [47]. These examples highlight how targeting shared mechanisms can yield dual benefits for brain and heart health.

Emerging therapies are also drawing attention, particularly mitochondrial-derived peptides (MDPs), such as Humanin, MOTS-c, and SHLP1–6. These peptides play key roles in maintaining cellular homeostasis and have shown protective effects in both neurodegenerative and cardiovascular models [48]. Humanin and SHLP2 protect neurons by reducing Aβ toxicity and preserving mitochondrial integrity, while MOTS-c and SHLP2 enhance metabolic resilience, especially during aging, diabetes, and ischemic stress [48]. Their ability to function as mitochondrial stress responders and retrograde signaling molecules positions MDPs as promising candidates for future therapies.

Lifestyle interventions, such as exercise and diet, further support mitochondrial health by enhancing biogenesis, oxidative capacity, and epigenetic regulation [49]. Resistance training and caloric restriction have been shown to enhance mitochondrial bioenergetics, while certain dietary components like cruciferous vegetables boost mitochondrial DNA content, potentially offering additional therapeutic value [50]. These nonpharmacological strategies may complement drug-based approaches in a holistic treatment framework.

Despite this progress, clinical translation remains challenging. Mitochondria are ubiquitous, making targeted delivery complex and increasing the risk of off-target effects. The blood–brain barrier limits drug penetration into the central nervous system, and there is still a lack of reliable, noninvasive biomarkers to monitor mitochondrial health in real time. Individual variability and the long-term consequences of manipulating mitochondrial pathways also raise safety concerns. Future research must focus on overcoming these obstacles, particularly through clinical trials that rigorously assess the safety and efficacy of mitochondrial-targeted interventions. As our understanding of mitochondrial biology deepens, it may unlock new, integrative treatment strategies capable of addressing the shared underpinnings of both cardiovascular and neurodegenerative diseases. This approach holds the potential to improve patient outcomes and enhance quality of life for millions affected by these overlapping and debilitating conditions.

## Figures and Tables

**Table 1:** Key pathogenic pathways with mitochondrial involvement in cardiac dysfunction and neurodegenerative diseases (e.g. AD).

Pathways	In cardiac dysfunction	In neurodegenerative diseases (e.g. AD)	References
Diastolic dysfunction	Mitochondrial dysfunction impairs ATP production, reducing heart muscle efficiency and contributing to impaired blood-pumping ability and cerebral hypoperfusion.	Decreased ATP from mitochondrial impairment exacerbates cognitive decline due to reduced blood flow to the brain.	[8,9,29]
Cerebral hypoperfusion and stroke	Mitochondrial energy deficits exacerbate metabolic disruptions, leading to acidosis, oxidative stress, and cellular damage in heart failure.	Reduced cerebral blood flow leads to mitochondrial damage, promoting neuronal degradation and brain structural changes, such as atrophy.	[9,10]
Atrial fibrillation	Mitochondrial dysfunction contributes to irregular calcium handling and ROS production, increasing the risk of silent strokes and microinfarcts.	Leads to mitochondrial-driven oxidative stress and calcium imbalances, causing a higher risk of cognitive decline and dementia.	[11,13,14]
Protein aggregation	Mitochondrial proteotoxic stress from misfolded proteins impairs cell function and calcium regulation in cardiomyopathies.	Aβ and tau protein accumulation disrupts mitochondrial function, causing oxidative damage and cell death in neurons.	[8]
Mitochondrial dysfunction	Central to impaired fusion and fission balance, excessive ROS, and ATP deficiency, leading to cardiomyocyte death and heart failure.	Mitochondrial dysfunction disrupts energy production, increases ROS, and contributes to neurodegeneration and cognitive decline.	[31–33]
Oxidative stress	Mitochondrial ETC defects increase ROS production, leading to oxidative damage, reduced ATP, and impaired heart contractility.	Mitochondrial ETC dysfunction promotes neuronal death and accelerates the formation of Aβ plaques and tau tangles.	[31–35]
Shared genetic mutations (PSEN1, PSEN2)	Mutations affect mitochondrial function and promote protein aggregation in familial DCM and heart failure.	Genetic mutations linked to AD disrupt mitochondrial activity, affecting Aβ production and contributing to neurodegeneration.	[2,3]
Systemic amyloid deposition	Mitochondrial involvement in amyloid processing contributes to Aβ deposits in cardiac tissue, leading to arterial stiffness and compromised heart function.	Mitochondria contribute to amyloid deposition in cerebral vessels, worsening cerebral amyloid angiopathy and cognitive function.	[13,14]
Calcium dysregulation and overload	Excessive mitochondrial calcium causes mPTP opening, cell death, oxidative stress, and impaired heart contraction, contributing to cardiomyopathies.	Mitochondrial calcium overload contributes to synaptic failure, oxidative stress, and neuronal death, exacerbating cognitive decline and progression in AD.	[3,9,11,28]
Inflammatory signaling (MAVS)	MAVS dysregulation leads to excessive inflammation and oxidative stress, contributing to mitochondrial dysfunction and cardiac conditions like myocarditis and heart failure	MAVS activation triggers inflammation, oxidative stress, and impaired mitophagy. Its deficiency prevents mPTP-induced microglial activation and dopaminergic neuron loss.	[23]
Metabolic dysfunction (AMPK)	Reduced AMPK activity impairs mitochondrial function, leading to compromised ATP production, oxidative stress, and cellular injury. This contributes to ischemia-reperfusion injury and heart failure.	Impaired AMPK activity leads to mitochondrial dysfunction, reduced ATP production, and increased oxidative stress accelerating neuronal damage and Aβ accumulation.	[24,25]

Aβ, amyloid-β; AD, Alzheimer’s disease; AMPK, AMP-activated protein kinase; DCM, dilated cardiomyopathy; ETC, electron transport chain; MAVS, mitochondrial antiviral-signaling protein; mPTP, mitochondrial permeability transition pore; ROS, reactive oxygen species.

## Data Availability

No data were used for the research described in the article.
